# The CORE Group Polio Project: An Overview of Its History and Its Contributions to the Global Polio Eradication Initiative

**DOI:** 10.4269/ajtmh.18-0916

**Published:** 2019-10

**Authors:** Lee Losey, Ellyn Ogden, Filimona Bisrat, Roma Solomon, David Newberry, Ellen Coates, Dora Ward, Lisa Hilmi, Karen LeBan, Vanessa Burrowes, Henry B. Perry

**Affiliations:** 1 CORE Group Polio Project, Washington, District of Columbia;; 2 United States Agency for International Development, Washington, District of Columbia;; 3 CORE Group Polio Project/Ethiopia, Addis Ababa, Ethiopia;; 4 CORE Group Polio Project/India, Gurgaon, India;; 5 CORE Group, Washington, District of Columbia;; 6 Department of International Health, Johns Hopkins Bloomberg School of Public Health, Baltimore, Maryland

## Abstract

The CORE Group Polio Project (CGPP) has contributed to polio eradication by successfully engaging civil society, particularly the non-governmental organization (NGO) community. This engagement, which began with a grant from the U.S. Agency for International Development in 1999, has contributed to improvements in routine immunization programs, polio campaign quality, and surveillance for acute flaccid paralysis in many challenging geographic areas. The CGPP has worked closely with polio eradication partners in a collaborative and supportive role. The CGPP has focused largely on high-risk areas with marginalized or hard-to-reach populations where health systems and immunization programs have also been weak and where transmission of poliovirus had not been stopped. The CGPP has engaged local civic leaders and communities in ways to complement top-down vertical efforts of ministries of health and other partners in the Global Polio Eradication Initiative. The CGPP has developed innovative strategies to detect cases using community-based surveillance, promoted independent campaign monitoring, established cross-border initiatives, and developed a strong and creative cadre of community mobilizers to track missed children and deliver behavior change education. Many of the innovations and approaches that the CGPP helped to develop are now being replicated by governments and international agencies to tackle other public health priorities in underserved and marginalized communities around the world. This article is the first in a series of articles describing the work of the CGPP. Because the article describes the work of more than 40 NGOs in 11 countries over 20 years, it provides only an overview, leaving many important details and variations of the CGPP’s work to be described elsewhere, including in other articles included in this series.

## INTRODUCTION

Thanks in large part to the urgings of the Pan American Health Organization and Rotary International, the World Health Assembly of the WHO launched the Global Polio Eradication Initiative (GPEI) in 1988 when it was estimated that 350,000 children were still being paralyzed by polio each year. There are between 200 and 2,000 asymptomatic infections for every symptomatic case of paralytic polio. Accordingly, in 1988, there were probably at least 70 million cases of wild poliovirus (WPV) infection being transmitted annually throughout the world, and 125 countries were considered to have ongoing circulation of indigenous WPV. A decade later, in 1998, the number of clinically recognizable cases of polio had dropped by 95%, the region of the Americas was polio-free, and the Western Pacific and European regions were almost polio-free. Type 2 WPV was eliminated globally the following year in 1999.

Pan American Health Organization led the way by achieving polio-free status for the Americas in 1994,^1^ leaving the remainder of cases primarily in sub-Saharan Africa and parts of Asia. To accelerate the eradication of WPV in Africa and Asia, mass vaccination campaigns were initiated in the mid-1990s. At that time, the standard four doses of oral polio vaccine (OPV) given at birth and at 6, 10, and 14 weeks of age were considered sufficient to ensure immunity, and expert opinion led to the strategy of adding a few doses per year in mass campaigns in places where routine immunization coverage was low. This would presumably create the herd immunity needed to provide protection against transmission of WPV. These mass campaigns also had the added benefit of supplanting the endemic WPV with the live attenuated vaccine virus in the general population, thereby providing both direct and indirect exposure to the attenuated vaccine virus. The strategy was both simple and visionary, and it succeeded in interrupting the transmission of WPV in most countries—but not all. A great many non-governmental organizations (NGOs), including CORE Group members, participated in these early campaigns in the 1990s by providing logistical and mobilization support at the provincial, district, and community levels where they were already actively implementing health projects unrelated to polio.

By 1999, high levels of polio immunization coverage had led to the elimination of polio from all but a few areas of the world. At that time, a “Four Pillars Strategy” was developed that has continued to guide the GPEI to the present day, consisting of the following:

1. Strengthening of routine immunization services and expansion of immunization coverage in the population, including coverage with at least three doses of OPV
2. Supplemental immunization with OPV to build and sustain population/herd immunity
3. Outbreak response vaccination campaigns
4. Surveillance for acute flaccid paralysis (AFP)

However, at that same time in 1999, “pockets” of polio transmission had developed where there was a critical mass of children who had consistently remained insufficiently immunized and who provided a reservoir for WPV transmission. To completely interrupt transmission, an intensive effort was required to reach every child in these “pockets” multiple times. It had become clear that these “pockets” of WPV transmission were in mobile populations, slums, politically insecure areas, communities inhabited by religious or ethnic minorities, densely populated areas, and underserved populations that experience difficulty accessing health services.

The remaining cases were almost exclusively arising in these subpopulations. Furthermore, in socially marginalized populations, community resistance due to many years of frequent polio immunization campaigns became yet another obstacle to reaching these subpopulations. Anti-vaccine messaging led to increased refusals in some places, and lack of confidence and distrust contributed to hidden refusals. By 2001, there was a growing awareness that annual or even semiannual national mass immunization campaigns were insufficient to achieve the high levels of immunization required to interrupt transmission in these high-risk, hard-to-reach populations.

The interruption of WPV circulation ultimately depends on sufficiently high vaccination coverage to establish herd immunity. Although it was widely thought that herd immunity could be achieved with routine vaccination coverage rates of around 80%, experiences with polio eradication demonstrated that both the number of doses and the coverage rates necessary to interrupt transmission in high-density populations with poor sanitation and high levels of malnutrition and diarrhea had to be much higher. To succeed, a higher percentage of children needed more doses. Achieving this goal was additionally challenged by civil war, civil unrest, and insurgencies in countries such as Angola, Nigeria, South Sudan, Somalia, Afghanistan, and Pakistan. The quality of routine and campaign vaccination coverage—namely, the percentage of children vaccinated and the ability to detect cases through high-quality surveillance—has led to the elimination of WPV transmission throughout most of the world. Where the coverage is of high quality (meaning the percentage of the population being immunized is high), the WPV will not find enough susceptible children to sustain transmission. And, where case-based and environmental surveillance is strong, any remaining cases or silent transmission will be identified.

Consequently, the U.S. Agency for International Development (USAID) along with other major international donors and technical support groups such as the United Nations Children’s Fund (UNICEF) and the WHO came to recognize at that time the critical importance of identifying high-risk areas, developing focused social mobilization activities to increase the coverage of polio immunization (as well as the coverage of other basic immunizations), and implementing high-quality surveillance to identify new outbreaks. High-risk areas were defined as areas with low polio immunization coverage in combination with one or more of the following characteristics: 1) proximity to geographic areas with active WPV transmission; 2) a history of previous outbreaks in the area; 3) the presence of nomadic, mobile, or socially marginalized subpopulations; 4) inadequately functioning AFP surveillance systems; 5) the presence of insecurity, civil unrest, or armed conflict; or 6) a limited capacity to respond to outbreaks.

It was simply not enough to get the vaccine to the doorstep if the families refused to participate. Similarly, quality programming was difficult to achieve without reliable campaign coverage data. Both these factors underscored the importance of interpersonal communication and evidence-based communication strategies. Furthermore, numerous instances arose in which incorrect immunization campaign coverage figures were produced through official channels. Thus, it became critically important to develop independent campaign monitoring to measure the quality of coverage during campaigns. To stop polio transmission in these “pockets,” high-quality, current, and localized immunization coverage data were needed along with high-quality AFP surveillance to identify new outbreaks.

### The CORE Group.

The CORE Group (formally named CORE Inc.) is an association of NGOs working with communities around the world to improve health. Its mission is to “improve and expand community health practices for underserved populations, especially women and children, through collaborative action and learning,” and it envisions “communities where everyone can attain health and well-being.”^2^ The CORE Group evolved in the 1990s as a group of NGO health practitioners who were sharing best practices and ideas on how to implement USAID Child Survival projects, including approaches to behavior change education targeting mothers and their children. The CORE Group became a formal entity in 1997.

Since that time, the CORE Group has convened thousands of practitioners working in global community health to share knowledge, evidence, and best practices so that they can translate these into programs that can achieve a demonstrable impact on health.^3^ The CORE Group also hosts the International Community Health Network, a large global network of civil society organizations. CORE Group’s members include international NGOs with a long history of collaborating with civil society organizations and with marginalized and hard-to-reach communities around the world. The CORE Group Polio Project (CGPP) was established in 1999 to promote greater NGO engagement in community health, making it possible for USAID to channel grants to them to work on polio eradication in high-risk areas.

## THE FIRST DECADE OF THE CGPP (1999–2008)

In 1998, USAID met with the CORE Group board of directors to discuss NGO engagement in polio eradication. Based on these discussions, World Vision submitted a proposal to USAID on behalf of the CORE Group NGO members. In 1999, USAID gave the CORE Group a US$25 million award for 5 years, and a committee worked to search for the first project director. The CORE Group and USAID established guiding principles for the CGPP, including the need for the NGOs to work cooperatively in high-risk places that would benefit the most from the CGPP’s engagement. The first project director traveled to potential target countries to assess the need and feasibility of launching the CGPP. The CGPP issued an initial request for proposals to CORE Group members to work in the polio-priority countries of Angola, India, Ethiopia, Uganda, Bangladesh, and Nepal and invited the Ministry of Health (MOH), the WHO, UNICEF, Rotary International, donors, and NGOs to participate in a preliminary partners workshop to discuss the polio eradication context in that country, the current roles of various partners, and the potential contributions that NGOs might make. These workshops provided the opportunity to present the CGPP in an open and transparent manner, allowing for debate and discussion across a range of partners, organizations, and interests to ensure that what evolved was understood and supported by the larger polio eradication community.

The initial CGPP headquarters team worked with the secretariats to develop a strategy for how the NGOs should plan and design a polio project. The basic requirements for funding project proposals were as follows:

1. There should be a full description of the proposed project.
2. The project should be built around NGO collaboration.
3. One NGO should host the in-country secretariat, through which the NGOs in country would collaborate.
4. The MOH and other implementing partners should provide a letter of support.

The first countries to start a national CGPP were Uganda, Angola, India, Nepal, Bangladesh, and, later, Ethiopia. Endemic transmission of WPV ended in Uganda (1996), Nepal (2000), and Bangladesh (2000),^4^ and the CGPP closed its programs there. In each country, the CGPP collaborated closely with the USAID mission and the in-country Interagency Coordinating Committee (ICC) for polio eradication.† In addition, the CGPP developed agreements with the government in close collaboration with the WHO and UNICEF. The in-country CGPP focused on community-based surveillance and on expanding vaccination coverage through social mobilization, logistical support, planning, and independent campaign monitoring for both routine immunization services and special immunization campaigns.

This early experience set the stage for later CGPP project launches in Nigeria, Kenya, Somalia, South Sudan, and Afghanistan. Although the initial launch of CGPP in a new country was often met with a degree of resistance and skepticism from other in-country GPEI partners, over time the CGPP developed strong working relationships with other partners, including the MOH, WHO, UNICEF, Rotary International, and donors, contributing jointly to improved polio programming.

With financial support from USAID, the CGPP launched social mobilization down to the household level and provided technical support for grassroots programming in geographic areas at high risk of WPV transmission in Angola, Bangladesh, Ethiopia, India, Nepal, and Uganda. A CGPP secretariat was established in Angola, Ethiopia, and India to coordinate the in-country work. These secretariats became members of the in-country ICC.‡ The ICCs, chaired by the MOH, include the WHO, UNICEF, Rotary International, and major donors including USAID and, later, the Bill & Melinda Gates Foundation.

Various technical partners worked together to define high-risk areas of the country based on population immunity, previous campaign performance, known polio reservoirs, silent areas (where no reporting of AFP is presently occurring), refusals, insecurity, geographic inaccessibility, areas with border communities (i.e., at the edge of national boundaries), or the presence of large mobile or displaced populations. The WHO, UNICEF, and the CGPP secretariat coordinated, at the request of the MOH, to develop joint strategies for strengthening polio eradication activities in these selected high-risk areas and provide technical oversight, monitoring, and evaluation of activities of the CGPP’s NGO partners. From the start, the CGPP focused on improving the quality of campaigns through the collection of reliable coverage data, better micro-planning and logistical support, and strong community engagement to promote positive participation and acceptance on the part of the community members. NGO organization partners also carried out household surveys, analysis of survey data, and corrective programming.

### Development of the CGPP secretariat model.

Although NGO health projects often have a significant community-level impact in limited geographic areas addressing specific health concerns, their efforts can be somewhat isolated or balkanized and, therefore, not conducive to the achievement of large-scale global initiatives such as polio eradication. The CGPP used a secretariat model to establish better collaboration and coordination between numerous NGO partners (listed in Supplemental Appendix II) in polio eradication and between various levels of engagement from the community to the district, provincial, national, regional, and global levels.

In each CGPP implementing country, the CGPP sets up a secretariat office headed by a secretariat director who is supported by a small team of technical staff. The secretariat is hosted by one of the partner NGO sub-grantees but is tasked with maintaining a neutral, overarching responsibility for the implementation of the CGPP in that country by providing guidance, support, and oversight to all NGOs receiving sub-grants. In six of the seven currently active implementation countries, the CGPP gives approximately three major sub-grants to international NGOs, who in turn give out additional sub-grants to local partner NGOs for a total of approximately 10 sub-grants per country and a little more than 70 sub-grants for the CGPP globally. The NGO hosting the secretariat usually provides office space, and receives and disburses funds to support secretariat activities. Supplemental Appendix II contains a list of all the international and national NGOs that have participated in the CGPP. At present, the Afghanistan project is limited to a national coordinator who sits in the National Polio Emergency Operations Center and coordinates with provincial NGOs responsible for providing the government’s primary health care.

At the country level, the secretariat generally meets with the implementing partner NGOs on a monthly basis to ensure a high level of coordination and integration between the various NGOs and to ensure that each NGO is well informed about the national, regional, and global polio eradication guidelines, objectives, and indicators. The actual timeframe for meetings varies by country and whether or not there is a campaign to implement. The meetings with local implementing NGOs are also an opportunity for the NGOs to share lessons learned, raise concerns, and share their specialized knowledge of the communities they serve. The secretariat director presents the perspective of communities and civil society at national planning meetings, including the ICC meetings and Emergency Operations Center meetings.

As an active member of these planning meetings, the secretariat director is responsible for establishing strong multilateral communication both as a “spokesperson” for NGO/civil society perspectives and as a recipient of national and global policy decisions and a transmitter of these to the NGO partners. The secretariat director has both the opportunity and the responsibility to help influence national policy- and decision-making and to ensure that the partner NGOs follow national guidelines and plans. The NGO perspective provided a much-needed reality check on the planning process, especially around how communities would react to new initiatives or tactics.

The CGPP also established a global secretariat to oversee country secretariat teams and to represent NGO/civil society concerns at various regional and global planning meetings such as the Independent Monitoring Board (IMB) for Polio, the Polio Partners Group (PPG), various regional ICC meetings, and various Technical Advisory Groups (TAGs) for immunization and polio eradication. The global secretariat, based in Washington, DC, consists of a director, a deputy director (who is also the technical lead), a senior technical advisor for monitoring and evaluation, a senior technical advisor for communications, a project officer, a finance manager, and a contracts manager. The senior management functions are handled by the global director and the global deputy director/technical lead who select, supervise, and support the national secretariat directors, review and approve all sub-grants, maintain strong relations with CGPP donors (USAID and the Gates Foundation), and contribute to global planning and policy. From the start, the global secretariat was established as a virtual team, with staff coordinating from multiple cities and paid by different NGOs. This significantly reduced the headquarters operating costs and fostered ownership among partners.

### CORE Group Polio Project organizational strategies.

At the country level, the CGPP secretariat coordinates CGPP activities through contracts with international NGOs who are members of the CORE Group and are already working in-country. These international NGOs then contract with local or national NGOs in high-risk areas to carry out social mobilization and other activities to achieve the goals of polio eradication. The international and national NGOs that are interested in collaborating on polio eradication submit a bundled proposal in advance of receiving USAID funding. The bundled proposal describes where the NGOs will work, their implementation plan, and how they will collaborate with other partners. They also provide letters of support from the government, the WHO, and UNICEF, as well as letters of concurrence from the in-country USAID mission. The U.S.-based senior management team of the CGPP coordinates with USAID, World Vision (which hosts the global secretariat for the CGPP), the CORE Group, international NGOs, the WHO, UNICEF, Rotary International, the CDC, donors, and the country CGPP secretariats.

**Figure 1.**
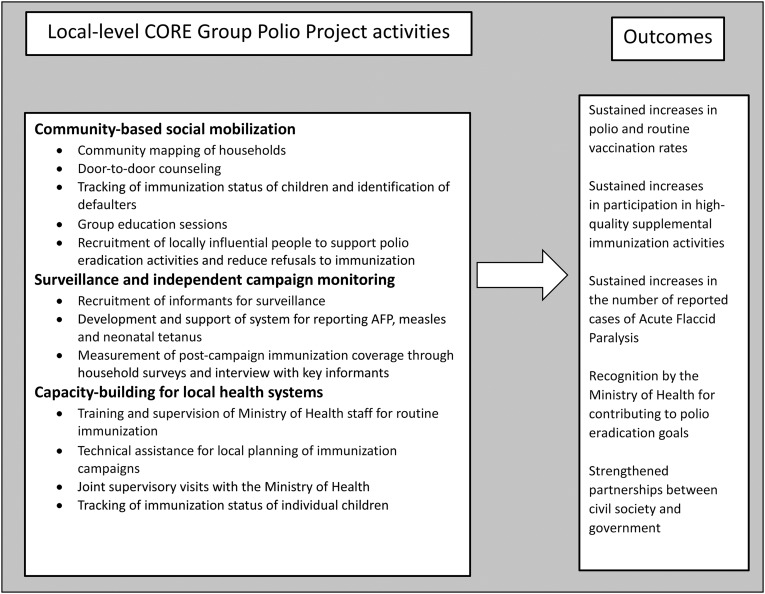
Overview of local-level CORE Group Polio Project activities, 1999–2008.

Examples of specific in-country activities of the CGPP that were carried out between 1999 and 2008 are shown in Figure 1. Community mapping of households and routine visits to all households made it possible to develop registries of pregnant women, register births, track the immunization status of newborns, trace defaulters, and ensure that they are appropriately immunized. The CGPP was also involved in national-level coordination of independent campaign monitoring, advocacy, and (in India) collaboration with the Social Mobilization Network (SMNet) partners. CGPP country partners developed short-term detailed work plans, which specified activities with specific locations and expected results. This approach made CGPP NGOs effective partners during polio immunization campaigns. CGPP supervisors supported field teams, identified field problems, and documented issues related to vaccine supply and household resistance. Whenever possible, NGOs shared training materials, job aids, reporting formats, and other materials, thus reducing duplication and overall costs.

From the start, one of the key strategies of the CGPP has been the utilization of community health workers, also sometimes called community mobilizers, to educate mothers and youth about the reasons for eradicating WPV and how transmission of poliovirus can be stopped. This strategy was a natural application and adaptation of the standard approach of NGOs to maternal and child health programming. In many cases, community health workers were already working on child survival projects managed by CORE Group member NGOs, conducting behavior change education in the community. Thus, adding polio messages to their existing activities required minimal adaptation. In Angola and Ethiopia, the community health workers were part-time volunteers, whereas in India, they received an honorarium. The decision to pay the community mobilizers in India was a joint decision with UNICEF. In Angola and Ethiopia, the NGOs chose not to pay the community health workers for fear of creating a non-sustainable expectation. CGPP health workers, NGO field staff, and government health workers sat together and studied district-level health data to determine where to canvas households and direct their mobilization efforts. The community health workers in some localities were also taught to conduct community-based AFP surveillance. NGO staff and community health workers were creative and persistent in putting into practice the commitment to reach every unvaccinated child in communities where there was endemic WPV or very low immunization coverage to decrease the build-up of susceptible cases and stop transmission of WPV.

The GPEI technical coordinating committees defined the districts and segments of districts in which the CGPP would work, and these areas were subject to change on short notice based on surveillance and immunization data and/or the presence of special high-risk and inaccessible populations. The international partner NGOs were already in-country, carrying out other projects. The polio eradication functions were integrated into their other ongoing field programs. Although international NGOs did not always have programs in the areas at high risk of polio transmission, they had the in-country expertise to identify local NGOs operating in these areas and to provide needed technical and administrative support. Local NGOs were the key to identifying local civic and religious leaders for cooperation and coordination.

By 2009, the CGPP had closed successful programs in Uganda, Nepal, and Bangladesh, although in 2018 the government of Uganda asked the CGPP to return and focus on refugees in the border areas with South Sudan. CORE Group Polio Project activities were ongoing in districts or portions of districts in Angola, Ethiopia, and India that contained a total population of 82 million people, including 27 million children aged 15 years or less. Among these were 3.8 million children in Angola, two million in Ethiopia, and 21.4 million in India. Between 1999 and 2008, the CGPP had trained 250,000 mobilizers (including community health workers, community leaders, and lay supporters in the community) to 1) provide essential, culturally relevant information about polio to mothers and caretakers; 2) promote good child immunization practices; 3) follow-up defaulters and track unimmunized children; 4) mobilize communities to support immunization campaigns; and 5) correct false information and dispel rumors about immunization campaigns. During this period from 1999 to 2008, the number of identified polio cases declined from 131 to three in Ethiopia, and from more than 1,000 to around 500 in Uttar Pradesh, India. Angola went from more than 1,000 in 1999 to zero from 2001 to 2005 and then had a reimportation of WPV from India in 2006, which lasted until 2011.

In addition to the specific activities noted earlier, there were several less tangible, less quantifiable results achieved by the CGPP by 2009. These included such actions as bringing together communities and elected leaders to address chronic problems in addition to time-sensitive polio eradication activities, a holistic approach to the newborn (such as the India “Butterfly Booklet” described below), micro-censuses and better population data (to use as denominators in calculating coverage at the local level), innovative approaches to nomadic and mobile populations, and engagement of religious leaders. Linking local community surveillance activities with the national MOH programs had a significant value as well.

In India, each new polio case would be a blot on administrative and health officials’ records. This compelled them to seek out catalysts such as CGPP mobilizers to start conversing with communities and their leaders, which further led to building bridges that went beyond acceptance of polio immunization to better access to government-provided rations, road repairs, and health camps.

**Figure 2.**
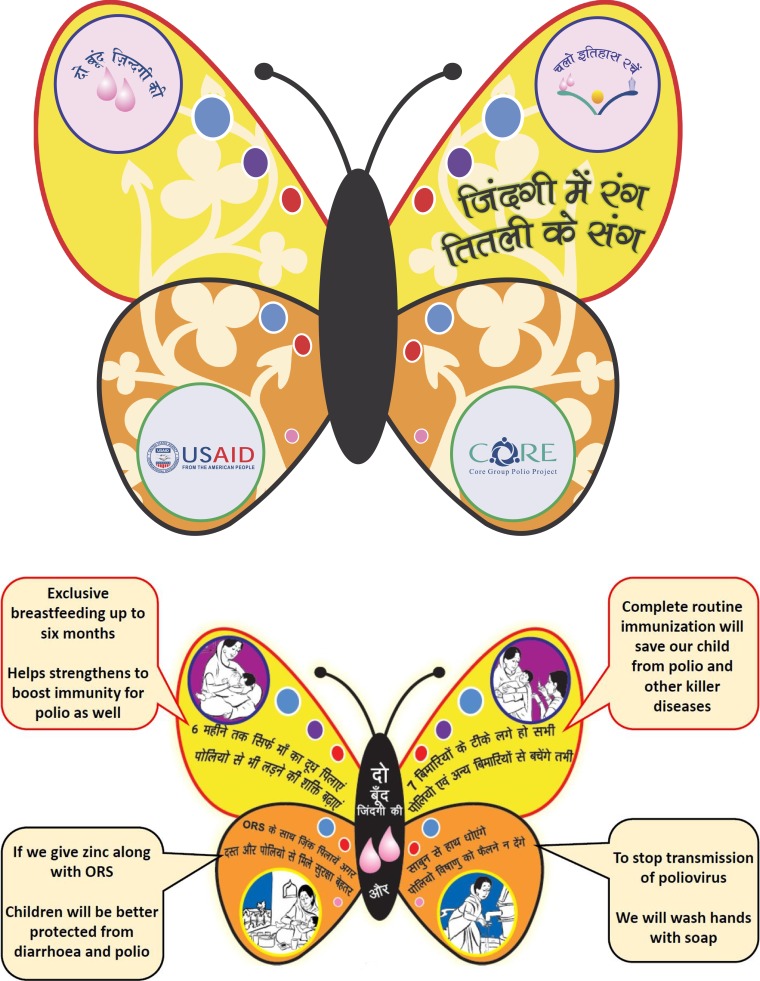
Drawings from the “Butterfly Booklet” developed for influential persons to promote elimination of poliovirus transmission in India.

The CGPP in India developed a “Butterfly Booklet” (see Figure 2) that was distributed to influential persons in the community to remind them of the importance of disseminating “polio plus” messages. It contains simple information explaining the links between polio eradication and exclusive breastfeeding, routine immunization, diarrhea management, and handwashing. Each of four charts in the booklet gives more detailed information on these four topics. The booklet enhances the messages that provide a rationale for why these four issues are important for polio eradication.

In Ethiopia, the CGPP developed and introduced community-based surveillance for AFP, measles, and neonatal tetanus in 2003 in collaboration with its partners, thereby filling gaps in identifying and reporting of these diseases. At that time, more than 2,000 community volunteers received training and were deployed to their respective *woredas* (districts). The use of community members for surveillance significantly increased the capacity for early detection of cases by expanding surveillance outside of health facilities. During the period from 2005 to 2008, a total of 119 suspected cases of AFP and 135 suspected cases of measles were detected and reported by CGPP community volunteers from 41 *woredas*.

### Development of a community engagement strategy.

A major contribution of the CGPP has been to expand program ownership to include the community by engaging community leaders and local influencers at the grassroots level. Before the onset of the CGPP, many CORE Group NGOs were already using community health workers to encourage behavior change in various maternal and child health projects. The CORE Group NGO partners who received USAID funding to assist in the GPEI applied this strategy, thereby creating synergies with their new polio projects and also adding polio messages to the other maternal and child health messages already present in their other projects.

In India, the CGPP collaborated with UNICEF to jointly establish the SMNet in the state of Uttar Pradesh to guide the coordination and collaboration of thousands of CORE and UNICEF community mobilization coordinators who had a very significant impact on community participation in polio campaigns through a well-designed behavior change education program. The success of this strategy in India promoted its adoption in other countries such as Nigeria, Pakistan, and Afghanistan.

India’s polio eradication program started as a booth-based mass campaign. Although it was successful, it still missed too many children. The government shifted to a house-to-house approach that reached more children, but this also led to increased suspicion about the government’s motives, given the unfavorable national experience with target-oriented family planning programs in the 1970s. CORE Group Polio Project’s observations and community feedback contributed to a more collaborative approach that emphasized child health more broadly and thereby served to increase transparency, build trust, and thus maintain national momentum toward polio eradication.

### Development of an approach to independent campaign monitoring.

One of the traditional and often controversial roles of civil society that the CORE Group has been able to play in polio eradication has been to challenge the status quo and push for greater transparency and innovation. One example of this was the push that the CGPP made for better data and independent polio immunization campaign monitoring to reliably measure the quality of individual campaigns and the population coverage achieved in Angola. At the start of the CGPP in Angola in 1999, the country suffered a major polio outbreak with more than 1,000 documented cases. Despite obvious evidence of poor vaccination coverage, the administrative data (based on reports submitted by health workers on the number of doses given) to estimate campaign coverage were routinely used to monitor campaigns, leading to official declarations that coverage had been more than 100%. In 2000, the CGPP advocated for and jointly implemented with the WHO, UNICEF, and the MOH the first rapid independent campaign monitoring survey (based on surveys of randomly selected households) and found that campaign vaccination coverage in Luanda (the capital of Angola) was only 71%. Although this finding was initially met with skepticism and resistance, in the long run, the importance of independent campaign monitoring was accepted. This contributed to the interruption of WPV circulation in Angola in 2001 by promoting much greater campaign quality.

### Achievements of the CGPP by 2008.

An evaluation^5^ of the first decade of CGPP activities concluded that the CGPP had made a strong contribution to global polio eradication efforts through its local community presence in high-risk areas, its training of local mobilizers to assist in polio eradication efforts, and its participation in polio eradication programming (particularly at the local level). Through its strong field presence, the CGPP was able to provide feedback to higher levels regarding the reality on the ground. The flexibility of CGPP field activities made it possible to quickly shift activities to new locations as the need arose. This greatly enhanced the CGPP’s effectiveness. The evaluation also found that the CGPP was increasingly valued over this period by other partners and stakeholders at the national level in polio eradication activities. The evaluation recommended that the country secretariats have a stronger funding base and more authority so that they could provide stronger oversight and technical support for field activities. Finally, the evaluation of that first decade made the following observation:

It is becoming clear to many who are working at top influential levels that there are huge benefits to have collaborating partners located within many small communities, a partner who is trusted, able to take the pulse of communities, learn citizen reaction to program efforts, knows the map of the community, [and] can head off rumors and diminish resistance to vaccines or child health care services.^5^

## THE CGPP: THE SECOND DECADE (2009–2018)

The second phase of the CGPP began in 2009 with financial support provided by USAID and supplemental funding provided by the Gates Foundation, which started in 2011. The main objectives in this second decade were to 1) build effective partnerships between NGOs, governments, and agencies involved in polio eradication; 2) support NGO efforts to strengthen immunization systems; 3) support NGO involvement in polio vaccination campaigns; 4) support NGO efforts to strengthen AFP case detection and surveillance for measles and neonatal tetanus; 5) support the timely documentation and use of information; 6) organize immunization activities in response to cases of vaccine-preventable disease identified through surveillance; and 7) support NGO participation in polio eradication certification activities.

Supplemental funding from the Gates Foundation was provided to the CGPP for the following specific objectives to 1) support campaign quality, routine immunization, community-based AFP surveillance, and independent campaign monitoring (in South Sudan); 2) decrease resistance to polio vaccination among caregivers of children aged 0–59 months (in Uttar Pradesh, India); 3) reduce the pool of susceptible or under-immunized children aged 0–59 months (in Angola); and 4) increase the availability of data to facilitate evidence-based decision-making that will strengthen polio eradication programs (in Angola, Ethiopia, and India).

The Gates Foundation funding to decrease resistance to polio vaccination in Uttar Pradesh (India) was specifically for providing other services beyond polio immunization among underserved populations, where basic health services were also weak. These populations had been growing weary of repeated polio campaigns with no apparent value to them while their basic health needs remained unmet.^6^

Global, regional, and national levels of the GPEI came to recognize that local community support had become a critical ingredient for polio eradication in high-risk areas. This had proven difficult to achieve in areas where other health problems were far more pressing, where there was suspicion about the motives of the GPEI, and where there was often resentment about lack of access to and ineffectiveness of government health services. Thus, Gates Foundation funding supported health camps in India and Nigeria for targeted high-risk groups where not only polio immunizations were provided but basic maternal and child health-care services, such as antenatal care, immunizations, and basic curative services, could also be provided. These camps also provided communities a chance to get acquainted with their health service delivery staff, and thus access to them was facilitated.^7^

In addition, analysis of surveillance of AFP for detection of possible polio cases showed geographic areas that were “silent” (i.e., no cases of AFP were detected or reported although background paralysis from non-polio causes were certainly occurring). In other areas, there were delays in case identification and investigation of AFP cases as parents of paralyzed children sought help from traditional or faith healers before making their way to a health facility. Lack of awareness of the need for reporting suspected AFP cases was common and a process for reporting cases directly from the community was not in place. The importance of community-based surveillance as an adjunct to the traditional facility-based AFP surveillance began to emerge as a priority around 2010.§

The Gates Foundation funding for India and Angola stopped in 2012 and 2014, respectively, but Gates funding continued to be the primary source of CGPP funding in South Sudan through 2018, where there is also a focus on community-based surveillance in the three difficult-to-access conflict states of Jonglei, Upper Nile, and Unity. In these settings, the CGPP reports most of the AFP cases.

The work of the CGPP during the second decade (2009–2018) was designed to be flexible so that international NGOs and their local NGO partners could “follow the virus” and respond to changing needs on the ground for support of polio eradication as defined by requests from the MOH and the Interagency Coordinating Committee. The CGPP gave greater emphasis to monitoring in Ethiopia and Angola by developing new quarterly monitoring forms and by conducting follow-up household surveys in 2013, 2015, and 2017. Supplemental Figures 1–3 provide illustrations of the field work carried out in Angola, Ethiopia, and Uttar Pradesh, India.

During this period, the CGPP demonstrated an ability to be flexible. It closed programs in Nepal, Bangladesh, and Angola following sustained interruption of WPV, and it launched new initiatives in Nigeria, South Sudan, Kenya, Somalia, Uganda, and Afghanistan in response to the needs of the GPEI. Although the CGPP discussed the continued use of the community-level workers with the governments as programs closed, CGPP’s programs were not designed for sustainability but rather for a targeted, time-limited surge for polio sustainability. Unfortunately, the networks of volunteer workers were not sustainable without supervision, funds, and oversight provided by the CGPP. There was also a concern about new outbreaks, but to date, WPV has not returned to any of the closed CGPP countries.

The CGPP also took on a much more visible global and regional role through participation in numerous global and regional meetings, such as the PPG in Geneva, the IMB, the Transition IMB, the Africa Regional Immunization TAG, the Horn of Africa TAG, the Strategic Advisory Group of Experts, the Global Alliance for Vaccines and Immunization, the civil society constituency, and various other groups as well. In addition, the CGPP generated technical reports, a polio toolkit, and peer-reviewed articles.‖^,8^ The CGPP secretariat model of collaboration and response has been adopted by other projects such as the Humanitarian Pandemic Preparedness project (2008–2011), sponsored by USAID, the CORE Group, the International Federation of the Red Cross, and FHI 360.

Published evaluations of the CGPP’s work in Ethiopia have demonstrated that the number of non-polio AFP cases reported annually in the CGPP catchment areas achieved the expected number within 3 years after initiating surveillance in 2004, and the Ethiopia CGPP has been able to maintain that level since.^9^ A later analysis demonstrated that by 2011, AFP surveillance in most of the CGPP catchment areas in Ethiopia had met international standards of quality.^10^

Published findings of evaluations of the CGPP activities in Uttar Pradesh, India, have documented the importance of mosque announcements in improved campaign performance in high-risk areas.^11^ The evaluations also found that in previously low-coverage, hard-to-reach, and resistant areas where SMNet was operating, the coverage was as high as or higher than in non-SMNet areas.^7,12^ In addition, the percentage of fully immunized children in the CGPP catchment areas increased from 48% to 63% between 2008 and 2011, whereas the percentage of fully immunized children in the entire state of Uttar Pradesh in 2011 was only 45%.^13^ A separate analysis also demonstrated that immunization coverage for the third dose of diphtheria, pertussis, and tetanus (DPT3) in the CGPP catchment areas in western Uttar Pradesh was substantially higher than that for the state of Uttar Pradesh as a whole.^7^

Analysis of household survey data from the CGPP catchment areas in Uttar Pradesh, India, and in Ethiopia demonstrated that children in homes that were visited by health workers had higher levels of routine polio immunization coverage than children whose homes had not been visited.^14^ Articles published in the peer-reviewed literature also describe in detail the community-engagement activities of the CGPP in India^7,11–13^ and Ethiopia.^9,15^

### Specific achievements of the CGPP cited in the 2017 evaluation.

Household surveys carried out in 2017 in CGPP implementation areas in India, Nigeria, Somalia, and Ethiopia revealed that community health workers trained and supported by NGOs were uniformly well-respected, knowledgeable, and influential in convincing parents to seek immunization, and that they were the top source of health information in hard-to-reach communities.^16^ They were the primary source of knowledge about polio and polio immunization campaigns in CGPP implementation areas, and their credibility and influence have grown over the past decade.

These surveys also revealed that routine immunization coverage, including that for the third dose of OPV, had increased in the CGPP target areas and had exceeded the national level. The increases in the percentage of children aged 12–23 months who were fully immunized were particularly notable in Nigeria (from 33% at baseline to 57%) and in Ethiopia (from 25% at baseline to 44%). The tracking of newborns and education of parents about the importance of the birth dose of OPV made it possible to achieve an OPV birth dose coverage of 56% in Ethiopia and 79% in India.^16^

The CGPP has supported supplemental immunization activities through monitoring, planning, social mobilization, and logistical support, with activities varying by country. The percentage of children younger than 5 years who were missed in each supplemental immunization activity in Nigeria decreased from 4.5% to 1.5%. In India, the percentage of houses missed decreased from 5.9% to 4.4%.

Community-based AFP surveillance carried out by the CGPP in South Sudan, Ethiopia, Kenya, Somalia, and Nigeria has contributed to the goal of identifying at least two cases of non-polio AFP per 100,000 children younger than 15 years within 14 days of the onset of paralysis. Early detection of AFP cases by community informants has also contributed to improving stool sample adequacy in remote areas in the Horn of Africa. Despite challenges of long distances, lack of roads, and civil unrest, South Sudan achieved a stool adequacy rate of 66% following the CGPP’s introduction of community-based surveillance.

In South Sudan, as described further in this series,^17^ the CGPP has established a timely, accurate, and robust community-based surveillance system, and the post-campaign monitoring conducted by the CGPP is the primary tool for measuring the quality of campaigns there. In 2014, the Southeast Asia region which includes India was declared polio-free despite deeply entrenched social resistance to immunization campaigns. The secretariat developed innovative behavior change communication strategies for social mobilization efforts. The use of child registries provided solid, up-to-date data to improve and track routine immunization coverage rates and facilitated improvement in the timing and coverage of the OPV birth dose timing and tracking of immunization defaulters. These strategies have been used by the GPEI in areas where the CGPP is not working.

## ASSESSMENT OF THE CONTRIBUTION OF THE CGPP TO THE GPEI

According to several technical advisors for the GPEI, the most important lesson learned from the Initiative is the importance of communications and community engagement to mobilize social and community support for vaccination, and that this is one of the important legacies of the GPEI for future global health work.^18^ These authors go on to say:

*For decades leading up to eradication, building social support for vaccination has begun with a comprehensive and wide-reaching approach to generate mass public support for polio eradication. As vaccination rates increased and the proportion of missed children became increasingly confined to discrete social and socioeconomic groups, communication and social mobilization strategies were refined and targeted to reach the most vulnerable families…. Through this process of mobilizing communities large and small, the polio program has developed the expertise to overcome the logistic, geographic, social, political, cultural, ethnic, gender and other barriers to working with the most-marginalized, most-deprived, and, often, most-security-compromised children and communities*.^18^

According to the WHO,^19^ the GPEI’s endgame strategic plan for 2013–2018 had, more than any other global health program in history, accessed the “chronically unreached, marginalized and most vulnerable populations in the world.” The characteristics and innovations developed to build social support for vaccination that were highlighted by this WHO technical group include 1) the relentless pursuit of the missed child; 2) the identification of individuals, themes, and social pillars that can unify and motivate diverse population groups for a common goal; 3) the mobilization of communities house-by-house on a grand scale not only for polio immunization but also for other discrete health interventions, such as vitamin A supplementation, measles vaccination, anti-helminthic administration, and distribution of soap, bed nets, and oral rehydration solution packets; 4) the creation of detailed local neighborhood vaccination team “micro-plans,” maps, and identification of locally influential people to assist in addressing those who are hesitant or resistant to immunization; 5) the tracking and counseling of pregnant mothers and follow-up of newborns for postnatal polio vaccination and routine immunization; 6) the collection and analysis of social data at the most local level to understand and engage effectively with the local population; 7) the tracking of mobile and migrant groups and communicating with these groups while they are in transit; 8) the engagement with groups while they are away from home during campaign days, such as with those attending social, cultural, or religious events (usually at weddings, shrines, or festivals); 9) the use of traditional, religious, community, and civil society leaders and structures for community mobilization; 10) improvements in interpersonal skills, management, and motivation of frontline health workers; 11) the development of evidence-based approaches to guide social mobilization and community engagement through ongoing, rigorous monitoring and evaluation; 12) the capacity to respond to community demands for additional services beyond polio immunization; and 13) assistance with vaccine distribution and maintenance of the cold chain.

All of these elements described above are key elements of the CGPP, and the CGPP has played a key role in developing and operationalizing these elements. But perhaps even more importantly, these innovations and approaches are now a global resource that can (and should) be applied to other global health priorities among underserved and marginalized groups around the world. This theme is explored further in a companion article in this series.^20^

Although the contributions of the NGOs varied from country to country, they brought a few critical components to the polio eradication mix: the capacity to produce high-quality immunization coverage data to evaluate campaign quality, the introduction of community-based surveillance to enhance facility-based surveillance, and improved behavior change education through the deployment of community social mobilizers. This can all largely be attributed to four strengths of NGOs: 1) they are present and active at the community level; 2) they are innovative and willing to take on new activities; 3) they have a commitment to producing and sharing high-quality, reliable data, even if it conflicts with data obtained through official governmental channels; and 4) they are willing to be accountable to their donors and to their communities.

## CONCLUSION

The CGPP has contributed to polio eradication by successfully engaging civil society, particularly the NGO community. This engagement has led to improvements in polio immunization campaign coverage, campaign monitoring data, and surveillance for AFP in many challenging geographic areas. The CGPP has collaborated with the international NGO community and local NGOs in high-risk areas to support campaigns, to strengthen routine immunization services, and to carry out AFP surveillance. These high-risk areas have almost always been in marginalized or hard-to-reach populations where health systems and their immunization programs have also been weak. The CGPP has engaged local civic leaders and communities in ways that complement top-down vertical efforts of MOH and other partners in the GPEI. Many of the innovations and approaches that the CGPP helped to develop are now being replicated by governments and international agencies to tackle other public health priorities in underserved and marginalized communities around the world.

## Supplemental appendix and figures

Supplemental materials

## Footnotes

† Interagency Coordinating Committees or their equivalent have been formed at the country level to improve coordination among partners in support of immunization programs and the control of vaccine-preventable diseases.

‡ In India, the Operations Group and Social Mobilization Working Group were structures used extensively for strengthening coordination. The director of the CGPP secretariat represented the CGPP by serving as a member of this working group.

§ Potential polio cases are identified through surveillance for AFP. In the absence of WPV, at least 2 cases of AFP per year would be expected in a population of 100,000 children aged less than 15 years. These are cases of acute onset of paralysis or limb weakness due to infection caused by a variety of different viruses or an inflammation of the spinal cord. Adequate AFP surveillance requires an identification of at least two AFP cases each year per 100,000 children aged less than 15 years. All identified cases are investigated. Stool specimens are collected and sent for laboratory examination to determine whether poliovirus is present. Adequate quality of AFP surveillance is defined as an annualized non-polio AFP reporting rate of ≥ 2 cases per 100,000 population aged younger than 15 years, and at least 80% of stool specimens from AFP cases are adequate for analysis.

‖ See Appendix I for a listing of all peer-reviewed articles published so far about the CGPP.
