# Supplementary material for: The CORE Group Polio Project: An Overview of Its History and Its Contributions to the Global Polio Eradication Initiative
Source: Am J Trop Med Hyg. 2019 Oct;101(4 Suppl):4–14. doi: 10.4269/ajtmh.18-0916 (PMC6776098; doi:10.4269/ajtmh.18-0916)
Supplement: Supplementary file 1 [file tpmd180916.SD1.pdf]

The following are supplemental materials and will be published online only

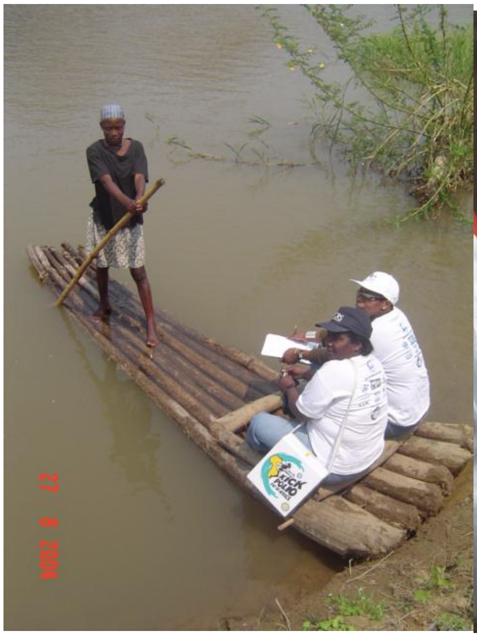

Transporting vaccine during a campaign to remote border communities inaccessible by road

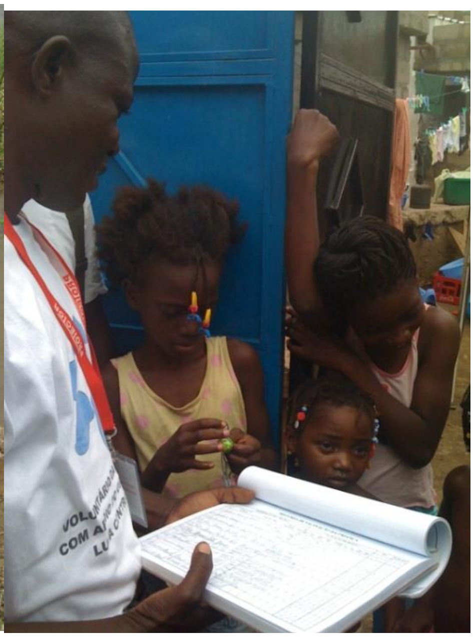

A volunteer and his vaccination registry

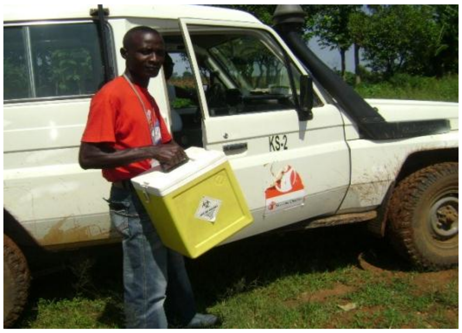

A CGPP partner transporting a specimen in Angola

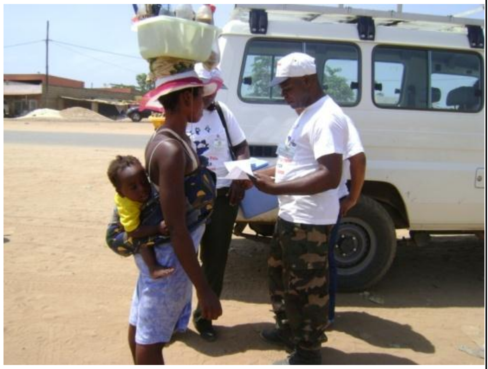

A volunteer conducting a survey for campaign quality monitoring

Supplemental Figure 1. CORE Group Polio Project activities in Angola

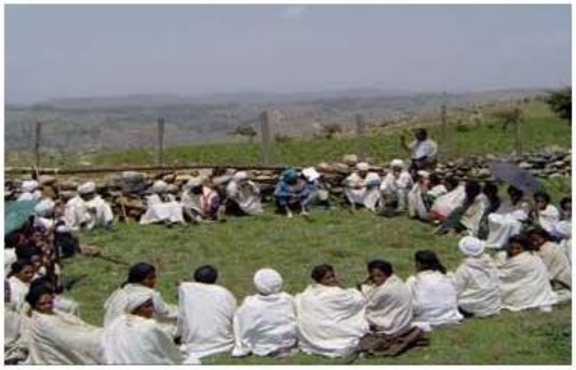

A group education session

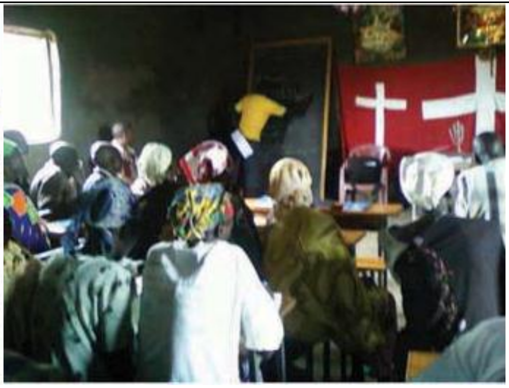

Training to integrate vaccination and polio messages into routine church messages in Gambella

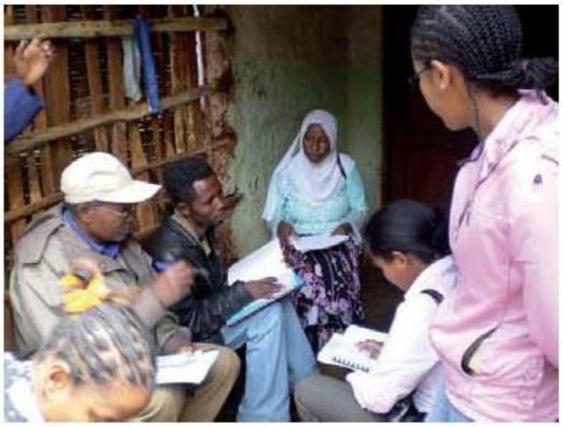

Volunteers enumerating households

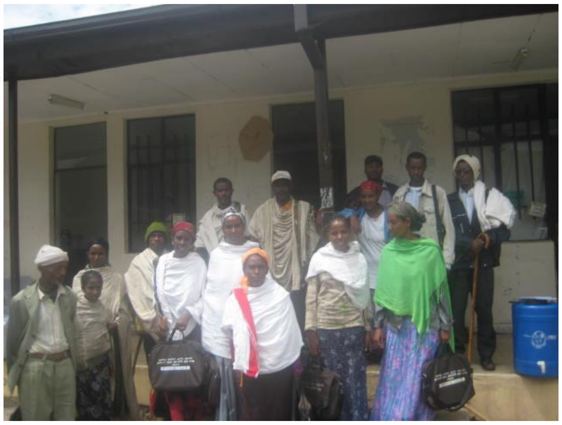

Volunteers at a monthly review meeting

Supplemental Figure 2. CORE Group Polio Project activities in Ethiopia

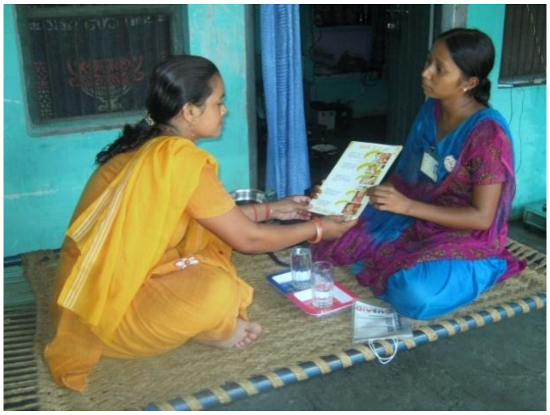

Interpersonal communication session by a Community Mobilizer

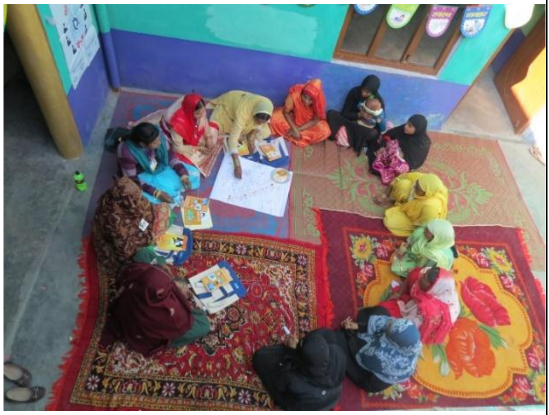

A group meeting conducted by a Community Mobilizer

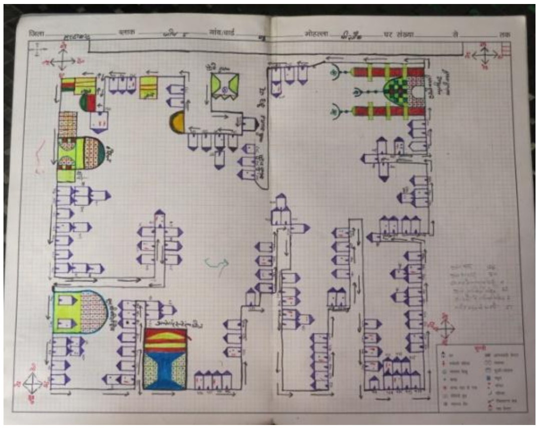

Area map of households prepared by a Community Mobilizer

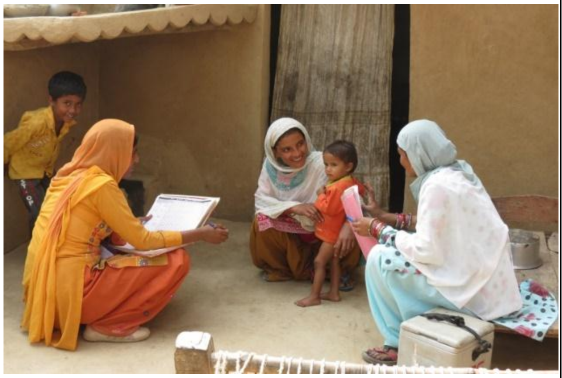

Home visit by a vaccination team along with a Community Mobilizer

Supplemental Figure 3. CORE Group Polio Project activities in Uttar Pradesh, India
