# Supplementary material for: The CORE Group Polio Project: An Overview of Its History and Its Contributions to the Global Polio Eradication Initiative
Source: Am J Trop Med Hyg. 2019 Oct;101(4 Suppl):4–14. doi: 10.4269/ajtmh.18-0916 (PMC6776098; doi:10.4269/ajtmh.18-0916)
Supplement: Supplementary file 2 [file tpmd180916.SD2.pdf]

The following are supplemental materials and will be published online only

Supplemental Appendix II. NGOs that have participated in the CGPP

Supplemental Appendix II. Table 1. International NGO partners of the CGPP, past and present*

International NGO	Country(ies) in which the NGO collaborated with the CGPP
Africare	Angola, Ethiopia
ADRA (Adventist Development and Relief Agency)	Ethiopia, India, Kenya, Nepal, Somalia
AMREF	Ethiopia, South Sudan
ARC (American Refugee Committee)	Kenya, Somalia, South Sudan
CARE	Angola, Bangladesh, Ethiopia, India, Nepal, South Sudan
CCF (Christian Children's Fund, now Child Fund)	Ethiopia, India
CRS (Catholic Relief Services)	Angola, Ethiopia, India, Kenya, Nigeria
IMC (International Medical Corps)	Nigeria
IRC (International Rescue Committee)	Ethiopia, Kenya, Uganda
Medical Teams International	Uganda
PCI (Project Concern International)	India
Public Health Services System (PHSS)	Nigeria
Plan	Bangladesh, Ethiopia, Nepal,
Salvation Army	Angola
Save the Children	Angola, Bangladesh, Ethiopia, Nepal, Nigeria, South Sudan
World Vision	Angola, Bangladesh, Ethiopia, India, Kenya, South Sudan

*****The names of the international NGOs that work with the CGPP in Afghanistan have been withheld for security purposes.

Supplemental Appendix II. Table 2. National NGO Partners of the CGPP, past and present.*

COUNTRY	NATIONAL NGO PARTNER			
**Angola**	Ãmmar	Assoder	Caritas	Twayovoca
**Bangladesh****	CARE/Bangladesh	Plan/Bangladesh	Save the Children/Bangladesh	World Vision/Bangladesh
**Ethiopia**	Alem Tena Catholic Church	CCRDA	EECMY	EOC
	Grarbet Ledekuman	HCS	OWDA	PCAE
	WASDA	WeSMCO		
**India**	Adarsh Sewa Samiti	ADP Ballia World Vision India	Agra Catholic Diocese Samaj Seva Sanstha	Akhil Bhartiya Gramodyog Sewa Sansthan
	All India Women's Conference	Arpan Gramin Vikas Samiti	AWARD	BADLAO
	Bahraich SDA School	Balia Gram Unayan Samiti	Bhartiya Kisan Sangh	Bhawani Siksha Prasar Parishad
	Carmel School and Hostels	Centre for Integrated Human Development	Clara Swain Mission Hospital	Daud Memorial Christian Gramin Vikas Samiti
	Diocese of Varanasi Social Welfare Society/Amarvani School for Hearing-Impaired	DISHA Children's Program	Don Bosco Centre	Dorma Ursline Convent Girl's School
	Giffard Memorial Hospital	Gorakhpur Environmental Action Group	Gramin Seva Sansthan	Gramodaya Seva Ashram
	Great Public Social Welfare Trust	GVK FHP	Innovative Approach for Social Development Society (IASDS)	Jai Prabha Mahila Vikas Kendra
	Jan Kalyan Samiti	Jan Manas Vikas Sansthan	Jan Priya Seva Sarnsthan	Janpragati Sansthan

COUNTRY	NATIONAL NGO PARTNER			
**India (continued)**	Jeevan Jyothi Community/BCM Hospital	Jeolikote St. Anthony's Project	Kalyan	Kolhan Mahila Sangathan
	KSRA	Lokmitra	Mahila Jagriti Seva Samiti	Malik Educational and Welfare Society
	Mamta Samajik Sansthan	Manav Seva Sansthan 'SEVA'	Manju Mahila Gramodyog Sewa Sansthan	Mattison Memorial Hospital
	Meerut Sewa Samaj	Methodist Girls' Inter College	Milan Sangha	Moradabad SDA School
	Mukti Niketan	Nav Bharathiya Nari Vikas Samithi	NBJK	Noatoli St. Ursula Project
	PANI	Param Jagrit Gramin Vikas Mandal	Parivar Vikas	Poorvanchal Gramin Vikas Sansthan
	Queen Mary's School, Delhi	Ram Manohar Lohiya	Ranchi SDA Hospital	Samiksha
	SANSKAR	Sarathi Development Foundation	Sarvodaya Seva Ashram	Shanti Nagar Hospital
	SHARD	Shivshakti Gramudyog Sansthan	Social Welfare Centre, Sundil	Society for All Round Development (SARD)
	Solanki Gramodyog Sewa Samiti	Subash Memorial Manav Uthan Evam Sewa Sansthan	Surat Hospital Trust	Swami Kalyananand Samaj Kalyan Shiksha Samiti
	SWARG	Ursuline Convent Girls' School Hostel	Uttar Pradesh Gramin Mahilia Vikas Parishad	Vigyan Foundation
	Vikas			
**Kenya****	ADRA/Kenya	CRS/Kenya	IRC/Kenya	World Vision/Kenya
**Nepal****	ADRA/Nepal	CARE/Nepal	Save the Children/Nepal	

COUNTRY	NATIONAL NGO PARTNER			
**Nigeria**	Federation of Muslim Women Association of Nigeria (FOMWAN)	Archdiocesan Catholic Healthcare Initiative (DACA)	Community Support and Development Initiative (CSADI)	Family Health and Youth Empowerment Organization
	Healthcare and Education Support Initiative (HESI)	Network for Integration & Rural Advancement (NIRA) or Community Development Foundation	WAKA Rural Development Initiative	Yetim Care Foundation (YETIM)
**Somalia**	Somali Aid			
**South Sudan**	Bio Aid	Catholic Diocese of Torit	Children Aid of South Sudan (CASS)	Christian Mission for Development (CMD)
	Community Aid for Development (CAD)	Live Well South Sudan	Nile Hope	SPEDP
	UNKEA			
**Uganda****	IRC/Uganda	Medical Teams International/Uganda		

* The names of the local and national NGOs that work with the CGPP in Afghanistan have been withheld for security purposes.

** In Bangladesh, Kenya, Nepal, and Uganda, these international NGOs contracted with their local or national affiliates to conduct activities in the CGPP implementation areas.
