# Supplementary material for: The CORE Group Polio Project: An Overview of Its History and Its Contributions to the Global Polio Eradication Initiative
Source: Am J Trop Med Hyg. 2019 Oct;101(4 Suppl):4–14. doi: 10.4269/ajtmh.18-0916 (PMC6776098; doi:10.4269/ajtmh.18-0916)
Supplement: Supplementary file 3 [file tpmd180916.SD3.docx]

**Supplemental Appendix 1. Peer-review Journal Publications about the CGPP**

1. Abraham K, Bisrat F, Fantahun M, Asres M, Kidane L, Rogie B, 2013. Acute flaccid paralysis surveillance status and community awareness in pastoralist and semi-pastoralist communities of Ethiopia. *Ethiop Med J 51 (Suppl 1).*
2. Asres M, Bisrat F, Kebede Y, Asegedew B, Getachew, Fantahun M, 2013. Knowledge and practice of frontline health workers (health extension workers and community volunteer surveillance focal persons) towards acute flaccid paralysis (AFP) case detection and reporting in pastoralist and semi-pastoralist areas of Ethiopia. *Ethiop Med J 51 (Suppl 1).*
3. Asres M, Fantahun M, 2013. Health facility preparedness for routine immunization services in Gambella Region, Ethiopia. *Ethiop Med J 51 (Suppl 1).*
4. Asres M, Tessema F, 2019. Contribution of plastic bags to the retention of child immunization cards in Gambella Region and Assosa Zone, Benishangul-Gumuz Region, Ethiopia. *Ethiop J Health Dev 33 (Special Issue).*
5. Asres M, Wachiso A, Bisrat F, Tadesse T, Kidanne L, Asegdew B, Asress A, Tessema F, 2019. Immunization service providers’ knowledge, attitude and practice in primary health care units in pastoral and semi-pastoral areas of Ethiopia: CORE Group Polio Project. *Ethiop J Health Dev 33 (Special Issue).*
6. Beyene EZ, Worku A, Bisrat F, Fantahun M, 2013. Factors associated with immunization coverage among children age 12–23 months: the case of zone 3, Afar Regional State, Ethiopia. *Ethiop Med J 51 (Suppl 1).*
7. Bisrat F, 2019. Endeavors to improve immunization uptake in Ethiopia. *Ethiop J Health Dev 33 (Special Issue).*
8. Bisrat F, Abdissa S, Asres M, Tadesse T, Kidanne L, Asegdew B, Zeleke S, Asress A, Tessema F, 2019. Healthcare workers’ readiness to provide immunization services at primary health care units in pastoral and semi-pastoral regions in Ethiopia: Core Group Polio Project implementation areas. *Ethiop J Health Dev 33 (Special Issue).*
9. Bisrat F, Fantahun M, 2013. Contributing towards polio eradication in Ethiopia*.* *Ethiop Med J 51 (Suppl 1).*
10. Bisrat F, Kidanel L, Abraha K, Asres M, Dinku B, Conlon F, Fantahun M, 2013. Cross-border wild poliovirus transmission in CORE Group Polio Project areas in Ethiopia. *Ethiop Med J 51 (Suppl 1)*: 31–39.
11. Choudhary M, Solomon R, Awale J, Dey R, 2018. Demand-side determinants of timely vaccination of oral polio vaccine in social mobilization network areas of CORE Group Polio Project in Uttar Pradesh, India*. BMC Infect Dis 18:* 222.
12. Coates EA, Waisbord S, Awale J, Solomon R, Dey R, 2013. Successful polio eradication in Uttar Pradesh, India: the pivotal contribution of the Social Mobilization Network, an NGO/UNICEF collaboration. *Glob Health Sci Pract 1:* 68-83.
13. Curry DW, Bisrat F, Coates E, Altman P, 2013. Reaching beyond the health post: community-based surveillance for polio eradication. *Dev Pract 23*: 69-78.
14. Curry DW, Perry HB, Tirmizi SN, Goldstein AL, Lynch MC, 2014. Assessing the effectiveness of house-to-house visits on routine oral polio immunization completion and tracking of defaulters. *J Health Popul Nutr 32*: 356-66.
15. Dinku B, Bisrat F, Kebede Y, Asegedew B, Fantahun M, 2013. Knowledge of mothers on poliomyelitis and other vaccine preventable diseases and vaccination status of children in pastoralist and semi-pastoralist areas of Ethiopia. *Ethiop Med J 51 (Suppl 1).*
16. Dinku B, Kumie A, Bisrat F, 2013. Linking community volunteer surveillance focal persons with health extension workers on polio surveillance.  *Ethiop Med J 51 (Suppl 1).*
17. Kidanne L, Bisrat F, Dinku B, Lynch M, Fantahun M, 2013. Newborn tracking for polio birth dose vaccination in pastoralist and semi-pastoralist CORE Group Polio Project implementation districts (woredas) in Ethiopia. *Ethiop Med J 51 (Suppl 1).*
18. Kidanne L, Solomon M, Bisrat F, Asres M, Tadesse T, Asress A, Asegdew B, Zeleke S, Tessema F, 2019. Child vaccination timing, intervals and missed opportunities in pastoral and semi-pastoral areas in Ethiopia. *Ethiop J Health Dev 33 (Special Issue).*
19. Rogie B, Berhane Y, Bisrat F, 2013. Assessment of cold chain status for immunization in central Ethiopia. *Ethiop Med J 51 (Suppl 1).*
20. Tadesse T, Gelaw B, Haile Y, Bisrat F, Kidanne L, Asres M, Asress A, Asegdew B, Tessema F, 2019. Immunization service availability and readiness in primary health care in pastoral and semi-pastoral CGPP Ethiopia implementation districts. *Ethiop J Health Dev 33 (Special Issue).*
21. Tessema F, Kidanne L, Bisrat F, Asres M, Tadesse T, Asress A, Asegdew B, Zeleke S, Bederu N, 2019. Child vaccination coverage and dropout rates in pastoral and semi-pastoral regions in Ethiopia: CORE Group Polio Project implementation areas. *Ethiop J Health Dev 33 Special Issue.*
22. Weiss WM, Choudhary M, Solomon R, 2013. Performance and determinants of routine immunization coverage within the context of intensive polio eradication activities in Uttar Pradesh, India: Social Mobilization Network (SMNet) and CORE Group Polio Project (CGPP). *BMC Int Health Hum Rights 13: 25.*
23. Weiss WM, Rahman MH, Solomon R, Singh V, Ward D, 2011. Outcomes of polio eradication activities in Uttar Pradesh, India: The Social Mobilization Network (SMNet) and CORE Group Polio Project (CGPP). *BMC Infect Dis 11*: 117.
24. Weiss WM, Rahman MH, Solomon R, Ward D, 2013. Determinants of performance of supplemental immunization activities for polio eradication in Uttar Pradesh, India: social mobilization activities of the Social Mobilization Network (SMNet) and CORE Group Polio Project (CGPP). *BMC Infect Dis* *13:* 17.
